# Healthcare workers’ perceptions of how eHealth applications can support self-care for patients undergoing planned major surgery

**DOI:** 10.1186/s12913-022-08219-4

**Published:** 2022-06-30

**Authors:** Anna Granath, Kerstin Eriksson, Lotta Wikström

**Affiliations:** 1 Department of Nursing Science, School of Health and Welfare, Jönköping University, Box 1026, 551 11 Jönköping, Sweden; 2 Department of Anaesthesia and Intensive Care, Ryhov County Hospital, Jönköping, Sweden

**Keywords:** eHealth, Healthcare workers, Perceptions, Major surgery, Self-care

## Abstract

**Background:**

In planned major surgery the duration of inpatient hospital care during the last decade has decreased because of a combination of different perioperative interventions. It is expected that patients can manage the needed pre- and postoperative self-care to a large extent on their own. This entails challenges to healthcare system to deliver appropriate information to patients in a safe and efficient manner. The aim of this study was therefore to describe healthcare workers’ perceptions of how eHealth applications can support patients’ self-care in relation to planned major surgery.

**Methods:**

Semi-structured interviews were performed with sixteen healthcare workers from different disciplines. The interviews were transcribed and analysed using the phenomenography approach.

**Results:**

Healthcare workers perceived both positive aspects and challenges with eHealth applications for self-care. eHealth applications can work as an information source, affect patients’ understanding of self-care, improve patients’ participation in self-care, streamline communication with healthcare professionals and improve patient safety during the pre- and postoperative period. The challenges included perceptions of that eHealth applications may have negative impact on personal interaction in care. eHealth applications may not be useful to all patients because of lack of equipment or knowledge and may increase patients’ suffering if physical visits are replaced by digital solutions.

**Conclusions:**

This study improves our understanding of healthcare workers’ perceptions of how the use of self-care eHealth applications can support patients in performing pre- and postoperative self-care for major surgery. Access to appropriate and personalized information and instructions can improve patients’ understanding of self-care and enhance the participation and safety of those who can afford and handle digital tools. All these aspects must be considered in future digital development of eHealth applications to guarantee a person-centered care.

## Background

In recent decades, the duration of inpatient hospital care for planned major surgery has decreased. The reasons for this are the introduction of fast-track programs with multimodal approaches to care and the use of a combination of different perioperative interventions [1]. Due to shorter hospital stays, patients have to manage the pre- and postoperative self-care on their own [2]. World Health Organization defines self-care as “the ability of individuals, families and communities to promote health, prevent disease, maintain health, and cope with illness and disability with or without the support of a health worker ([3]p(8)).” Self-care entails challenges to the healthcare system regarding the delivery of appropriate care information to patients in a safe and efficient manner. For patients, it requires understanding, participating in and being responsible for self-care measures in both preoperative preparations and in the postoperative recovery period [2].

Patient empowerment and participation involves the rights and opportunities to influence and engage in decision-making related to the care process through a dialogue based on patients’ preferences and experiences and professionals’ expert knowledge [4]. For planned surgery, the recovery phase can be enhanced and accelerated by the active participation of all team members in the multidisciplinary team, including the patient, and is necessary for reducing the negative post-surgery effects such as wound infections, cardiorespiratory complications, pain, nausea and vomiting [1]. Patient participation is a strategy for achieving person-centred care, which provides increased patient satisfaction, improved concordance between care providers and patients on treatment plans [5], and more opportunities for patients to take responsibility for their self-care, which, in turn, leads to better health outcomes, fewer healthcare visits and hence a lesser burden on society [6].

As patients are expected to take greater responsibility for and participate in their pre- and postoperative self-care, the development of various digital eHealth services for facilitating access to information and communication has significantly accelerated and are constantly evolving. A research group mapping the concept of eHealth defined it as follows:

“e-health is an emerging field of medical informatics, referring to the organization and delivery of health services and information using the Internet and related technologies. In a broader sense, the term characterizes not only a technical development, but also a new way of working, an attitude, and a commitment for networked, global thinking, to improve health care locally, regionally, and worldwide by using information and communication technology” [7].

Digital solutions work as tools for exchanging important health information and monitoring symptoms and can increase interaction, collaboration and communication between patients and healthcare professionals. eHealth solutions can help patients take responsibility for their self-care and facilitate positive changes in health behaviours for preventing the onset of acute and even long-lasting diseases by improving adherence to physiotherapy exercises and daily self-care activities [8–10]. In addition, eHealth can reduce errors and risks in health care. Improving quality and safety in self-care supported by eHealth, can also reduce the overall costs associated with health care [11].

Digital solutions for follow-up interventions and self-care support have quickly gained in importance, even in pre- and postoperative care. eHealth applications have been developed to enhance patient participation and recovery monitoring in both day care [12], and fast-track surgery [13]. Some of the purposes are to increase the availability of information to the patient and to improve guidance through the surgical procedure [14]. eHealth applications can, in turn, enhance patients’ compliance to self-care procedures during the recovery period, which results in better outcomes, such as increased performance of daily self-care and physical activities, lower levels of postoperative pain and increased satisfaction and quality of life [13, 15].

Studies have been conducted on healthcare professionals’ experiences of and attitudes towards the use of digital solutions in health care and home follow-up care [16–19]. Attitudes are a significant factor in eHealth’s acceptance and its efficiency of use and function [20]. It can also be a source of knowledge for future design of digital support for self-care within surgery. However, to the best of our knowledge, there are no studies on how healthcare professionals perceive the support via eHealth applications in terms of patients’ self-care in pre- and postoperative care related to major surgery. Therefore, the aim of our study was to describe healthcare professionals’ perceptions of how eHealth applications can support patients’ self-care in connection with planned major surgery.

## Methods

### Design

To achieve our main aim, we carried out a descriptive qualitative study with a phenomenographic approach. Phenomenography aims to describe differences in the way that people experience reality and perceive phenomena [21]. Using the first-order perspective, researchers are interested in how something really is. According to the second-order perspective, the focus is on how persons perceive a phenomenon [22]. Our study, which adopted the second-order perspective, focused on the different ways in which people make sense of eHealth applications’ supporting role for patients undergoing major surgery. To our study, we defined major surgery as planned inpatient surgery.

### Participants and settings

To cover a diverse range of genders, ages, professions, and work experiences with surgical inpatients, we purposefully and strategically recruited Swedish-speaking healthcare workers. Contact was made with identified representatives in different levels in the surgical or orthopaedic organisations working in either a county or university hospital or in a primary care setting in the southwest of Sweden. Information about the study and a request to participate was sent via email by the first author to these strategically selected healthcare workers. After receiving participation consent, we arranged time and place for the interviews. One person who agreed to participate was rejected because the daily services were mainly centred on day surgery. We arranged 17 interviews, but one participant chose to withdraw.

The participants’ median age was 49 years (30–66 years), and their median professional experience was 21 years (0.5–39 years). Most of the participants were female nurses, which correlates with the employee distribution in Swedish health care [23]. Seven of the nurses had specialist training in surgical, intensive, or primary care. Seven of the participants held leading healthcare positions, such as care unit manager, regional operations manager, deputy director and regional or local development strategist. Three of the nurses worked as contact nurses at an outpatient clinic. The participants’ characteristics are presented in Table 1.

**Table 1:** Participants’ characteristics

Participant characteristics	Participants (*N* = 16)
**Age**	
20–39	4
40–49	4
50–59	5
60 and above	3
**Gender:** Women / Men	13 / 3
**Professional affiliation**	
Nurses:^a^	
-Surgical care	4
-Orthopaedic care	7
-Primary care	1
-Development manager	1
Physicians:^b^	
- Specialist in surgery	1
- Specialist training in primary care	1
Physiotherapist orthopaedic setting	1
Strategist	1
**Setting**	
Primary care	2
County hospital	
- Surgical care	6
- Orthopaedic care	5
University hospital	
- Surgical care	3
**Professional experiences in health care**	
< 1–10 years	2
11–20 years	6
21–30 years	5
> 30 years	3

^a^of which five were in manager position

^b^of which one was in manager position

At the participating clinics, the perioperative care process begins with a patient’s enrolment visit to the clinic a couple of weeks prior to the planned day of the major surgery. During the visit, oral and written information is provided by the surgeon and the contact nurse. If required for medical reasons, a physiotherapist, and an anaesthesiologist exchange information with the patient. The information given to the patient includes preoperative preparations at home before surgery such as hygiene, food intake and possible adjustment of ongoing medication, further about the surgical care at the hospital and postoperative self-care measures at home such as pain relief and the importance of food intake and tailored exercises. Patients are often advised to visit the national online Healthcare Guide platform [24] for further information and access to their medical records. Patients are admitted the day before or on the day of the surgery and are discharged as soon as the medical condition allows it. At discharge, patients receive further information about the management and treatment of pain, wound dressing, post-surgical exercises and how and when to contact the clinic or the emergency department. After certain surgical procedures, a postoperative follow-up telephone call or out-patient visit is booked with a nurse, a surgeon and/or a physiotherapist to control and guide postoperative recovery. The contact with the surgical clinic is terminated when the patient’s recovery process has proceeded as expected and without any complications. Follow-up take place in primary or outpatient care, if needed, and varies due to surgery.

### Data collection

An interview guide with semi-structured questions, presented in Table 2, was designed by two of the authors who had experience with patients who had undergone major surgery and with phenomenographic research. A pilot interview was conducted to ensure that the questions provided answers that fit the purpose of the study. After a joint discussion, the questions were considered to adequately address the aim of the study. Therefore, the pilot interview was included in the analysis. In the interviews, the participants were asked to describe how eHealth in connection with inpatient surgery were perceived.

**Table 2:** Semi-structured interview guide

Mapping of opportunities/obstacles for eHealth in relation to inpatient surgery
-What are your perceptions of eHealth? Do you have any experience with eHealth from work or private?
-What are your perceptions of eHealth regarding communication with patients?
-What are your perceptions of how eHealth can be implemented in the pre- and postoperative care process for inpatient surgery?
-What aspects of self-care could be included in an eHealth application for inpatient surgery?
-Which patients would such an eHealth application be useful to?

A total of 16 interviews (15 by the first author and one by the third author) were conducted with healthcare professionals between May and December 2019 at the participants’ workplaces. Audio-recorded face-to-face interviews were conducted with one participant at a time, except for one interview when two participants were present because of their restricted work schedules. During the interviews follow-up questions were used to allow the participants to further develop their answers. Finally, all participants were asked if there was anything else that they wanted to add on eHealth in relation to major surgery. Interview length varied between 38 and 79 min (median length: 53 min). All interviews were transcribed verbatim. In the start of the project, we expected that up to 25 interviews were needed, but due to the outbreak of COVID-19 pandemic, the Swedish government banned unnecessary visits to care facilities, which meant that further interviews face-to face with healthcare workers could not be conducted.

### Data analysis

The analysis process followed the seven steps described by Sjöström and Dahlgren [22], (see Table 3). To become familiar with the content, we read the transcribed interviews several times. Based on the aim of the study, the most significant and meaningful elements in answer given by each informant were identified and condensed to focus on the essence of the content. Descriptions of the perceptions that were related to one another were then compared and grouped. After discussions in and revisions by the research team, the groups were finally divided into eight descriptive categories*.* This categorization of the data concluded the analysis and revealed the variations in the investigated phenomenon, which are presented in the following section and illustrated with quotations from the participants.

**Table 3:** The seven steps of data analysis according to Sjöström and Dahlgren [22]

Familiarisation	The 16 interviews that resulted in 406 pages (A4) of data were read and reread to become familiar with the material
Compilation	A total of 163 quotations corresponding to the aim of the study were found
Condensation	Quotations were condensed to retain only the essence of the content
Preliminary grouping of similar answers	Condensed quotations that dealt with similar perceptions were grouped and regrouped
Comparison of categories	The grouped perceptions were compared to find similarities and differences, which produced insights into the nature of the phenomenon. This categorization step was discussed by all the members of the research team
Naming the categories	Eight distinctly different categories were named with descriptive headings and divided into two main categories that were named with an adequate level of abstraction to reflect their essence
Contrastive comparison	The variations in every category were described in terms of resemblances and differences

## Results

All participants in this study had, for personal reasons, accessed their medical records via the web-based national eHealth service [24]. The majority had work-related experiences of digital technology and communication in health care. Three of the participants had received information on or had recently been involved in a start-up project for a digital application service for smartphones whose aim was to guide, inform, activate, and involve both patients and their relatives in the pre- and postoperative process. The other participants had never been in contact with eHealth applications in the context of surgical care. One of the participants was not familiar with the very concept ‘eHealth’ but was familiar with its features. The participants had different perceptions of what the term ‘eHealth’ means for them. During the interviews it emerged that the Swedish national web-based public health service was considered to be a form of eHealth. Associations were also made to company funded eHealth applications.

**Fig. 1:**
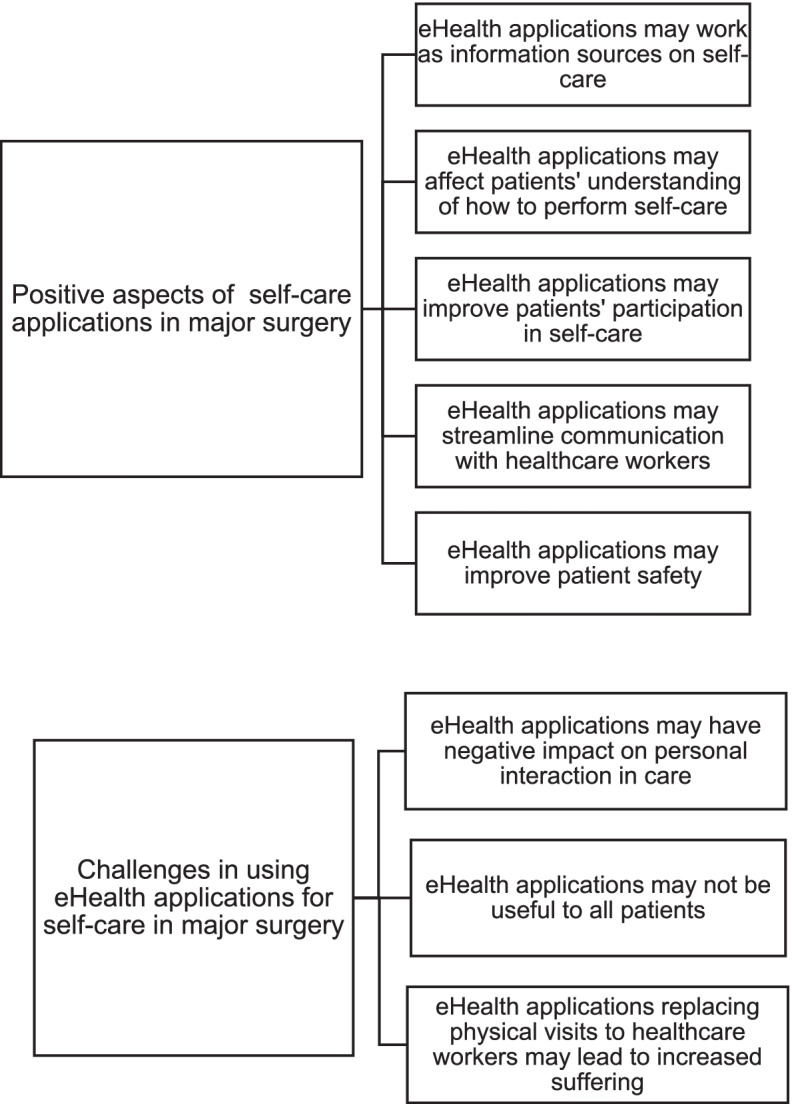
Results of analysis: Two main categories with associated subcategories

The analysis of the interviews resulted in two main categories with associated subcategories that describe the healthcare workers’ perceptions of how eHealth applications can support patients’ self-care in major surgery, see Fig. 1.

Perceived benefits were that an eHealth application could work as an information source about self-care and affect patients’ understanding of how to perform and participate in self-care. eHealth applications could also streamline the communication between patients and healthcare workers and improve patient safety. However, the healthcare professionals expressed concerns about challenges that eHealth applications may have negative impact on personal interactions, that they may not be useful to all patients and that it could lead to increased suffering if physical visits were replaced by eHealth applications.

### Positive aspects of self-care applications in major surgery

#### eHealth applications may work as information sources on self-care

The healthcare workers said they believed that in relation to planned major surgery, eHealth applications can work as a support for patients because such applications are constantly available and contain compiled and up-to-date information on self-care. eHealth applications could be more accessible if they did not require a personal login. Moreover, eHealth applications can provide necessary information about both own preoperative preparations and postoperative self-care interventions during the recovery period, which may make both patients and their relatives more prepared for and involved in the pre- and postoperative process.

“[In the app] there is all the information the patient needs: about the surgery and how to prepare oneself … and afterwards… He can watch the instructional videos as many times as he wants in the app, and he can read the text repeatedly … he doesn’t need twenty different papers; everything is in one place.” (Participant no 5, P5)

Digital applications were considered to serve as a complementary memory device to the information provided during the enrolment visit or hospital stay as exemplified in the following quotation:

“I do not think that the patient always remembers the information so well by someone telling … it is much easier to read and watch [the instructions] in peace and quiet at home.” (P11)

#### eHealth applications may affect patients’ understanding of how to perform self-care

It turned out that the information in eHealth applications should be designed to enhance patients’ understanding of how to perform self-care. Using standardised information, everyone can receive the same information, especially compared to oral information provided by different healthcare workers. However, standardisation was also considered to produce challenges in providing person-centred care. Therefore, the participants stated that there should be opportunities for customising the information in the application according to an individual patient’s needs and conditions to enhance patients’ understanding of how to perform self-care.

“We are different … someone is dependent on an emotional interaction [with healthcare professionals], another just wants a message … it is a big challenge for us to meet each patient and enable person-centred care, even in a standardised process.” (P5)

The healthcare workers said they perceived that eHealth applications could be more illustrative when combining informative texts, audio files, images and videos with real people that show how, for example, different exercises should be performed. In comparison with orally given information and information brochures, the given information in eHealth applications could also be tailored to be based on the individual patient’s situation and needs. This, in turn, should create and improve patients’ understanding of their self-care both before and after planned surgery.

“I would like to have eHealth information in pictures … to illustrate when during the day you should take medication, with a sun or a moon … images could serve as a complement to the text … someone may find it is easier with pictures than with text.” (P7)

One negative perception was that too much self-care information in eHealth applications could have negative consequences. For example, it was expressed that a patient may not understand comprehensive information, thus overlooking important parts of self-care.

“It is hard to include everything … it can be too much information [in an application], and then the patient would not read it.” (P1)

#### eHealth applications may improve patients’ participation in self-care

Self-care information and clear instructions were perceived important for patients’ participation in self-care management. The healthcare workers said they believed that reminders in the form of push notices and targeted individual messages could help and support patients. Information about the patient’s expected personal responsibility to follow the recommendations given by health care should be included in the application and together with reminders, this could also be motivating and increase the patient’s responsibility for, compliance to and participation in the self-care needed in the pre-operative preparations and post-operative recovery process. It was perceived as an advantage that relatives can also access the eHealth application and thus make them more involved.

“The patient can share the app with a relative … they can then also receive push notices … what is needed to prepare before surgery, to help and give support … (P8)

#### eHealth applications may streamline communication with healthcare workers

The healthcare workers said they believed that eHealth self-care applications in relation to pre- and postoperative care for major surgery may lead to more effective and time-saving communication and care for both patients and the healthcare system. For example, patients could easily find answers to frequently asked questions about self-care without burdening healthcare workers. Patients could also formulate their own questions about self-care and receive written answers. It was perceived that routines in health care need to provide updated information to patients, receive information and queries from patients and quickly deliver answers. One considered risk was otherwise that the response time could be higher compared to a phone call.

“You [the patient] will manage yourself at home, but you want quick contact with healthcare. I do not think you need to meet physically [every time for a follow-up] ... I can have a follow-up via the computer. The patient can show me a [digital] picture … he does not have to go to the hospital … he can live far away from here.” (P11)

Digital communication involving pictures, video calls and a self-reporting system was considered to work as a complement to physical visits to healthcare workers, thus facilitating patient assessment. By using video calls, more information about the patient could be obtained than via regular phone calls because one could also read patients’ body language and facial expressions.

“In video calls, you [the healthcare professional] may need to have a more active listening approach than an informing role. That is the demanding challenge … not what you have sent for information but for what you have received.” (P7)

#### eHealth applications may improve patient safety

Healthcare workers perceived that eHealth applications could increase the safety of patients’ self-care both pre- and postoperatively due to the availability of aggregated information from a controlled and reliable source compared to doing their own internet searches.

“It [the application] is always there … it offers security [to the patients] to have something to return to when we [the healthcare professional] are not standing next to them.” (P1)

Written information is usually only available in a limited number of languages, which was considered as a problem. Therefore, to avoid ensure patients’ self-care, an application should offer information in the patient’s and their close social networks native language. A self-care application should also include information on the negative side effects of drug treatments and what to do to prevent such effects; otherwise, patient safety could be impaired, contributing to a longer recovery period.

“[The patient] … must understand the instructions … otherwise, it can result in big problems…” (P3)

The healthcare workers said they believed that both automatic and self-written digital pop-up reminders about pre- and postoperative self-care interventions could contribute to self-care performed in a timely manner. Together with patients’ ability to tick off digital checklists before surgery (with items such as stopping to smoke, stopping to eat and drinking fluids, taking anticoagulants according to special prescription before the surgery, and checking that the skin is intact) and patients’ self-documentation of the ongoing postoperative recovery process, the digital system should automatically detect and warn of any pre- and post-operative complications in early stages and send new advice and information about upcoming self-care interventions. Ideally, the system should also alert health services when something is going wrong, and the patient needs to be contacted. The healthcare workers perceived that such features could enhance patient safety and are described in following examples:

“… [the patient] will get reminders [before surgery], such as “check that you have not received any wounds on your body,” … because then there can be no surgery … in this way, you will achieve a better security.” (P4)

“… information about when to make contact when there is a problem. We do not want to end up in a difficult situation. The app needs to contain information on early warning signals and when and whom to contact.” (P8)

### Challenges in using eHealth applications for self-care in major surgery

#### eHealth applications may have a negative impact on personal interaction in care

The healthcare workers said they believed that eHealth applications could work as a complement to other care interventions, but there were fears that such applications could replace the personal meetings between patients and healthcare providers, which often create meaning and satisfaction for both parts, and also diminish the holistic understanding of each patient’s situation. The healthcare workers claimed that eHealth applications cannot completely replace personal contacts:

“You cannot exclude conversations, dialogue, personal contact … then we promote the impersonal. We are social beings; we need to talk and not live through a digital device … You need the physical meeting that provides support and participation … you do not want to feel like a robot ... and a picture may not tell me the whole truth.” (P9)

#### eHealth applications may not be useful to all patients

The healthcare workers perceived that pre- and postoperative self-care information provided exclusively via eHealth applications may produce the risk that some patient groups could be excluded because of their lack of computer knowledge and skills in acquiring information or limited access to technical devices and internet connection. The elderly population was a patient group that was considered at risk of being excluded. Even users’ conditions, abilities, willingness, technical abilities, and attitudes to self-care in combination with the use of digital applications could affect self-care in a negative way.

“Everyone must keep up [with the digital development]. There is a group of older people today who have not really learned this … you see the shortcoming if they do not have someone who can help them, then they will be overlooked.” (P14)

#### eHealth applications replacing physical visits to healthcare workers may lead to increased suffering

If pre- and postoperative self-care information is provided mainly via eHealth applications or without the possibility of physically visiting healthcare workers, then, according to the professionals, there is a risk of increased patient suffering. Patients who are incapable of receiving or understanding digital information or those who choose not to contact the health service themselves to report any problems or complications that have arisen, may be exposed to increased suffering. Such concerns were considered to increase the risk of reduced quality of care.

“They [the patients] may never contact healthcare workers … they sit at home suffering and do not know that this is not how it should be.” (P12)

## Discussion

The main findings of this study reveal healthcare workers’ perceptions about how eHealth applications can provide both positive aspects but also challenges for the patient’s selfcare in connection with major surgery. Some of the benefits was that the availability of a self-care application could increase the awareness of and support patients’ pre- and postoperative self-care for planned major surgery. Healthcare workers believe that available and reliable information enhances patients’ understanding of self-care, which improves patients’ participation and patient safety. The use of digital self-care applications in relation to major surgery was also considered to create challenges by affecting the interactions between healthcare workers and patients in different ways. It was also considered possible to increase the risk of excluding different patient groups, especially those without digital knowledge or without access to internet services.

Healthcare workers believe that available, reliable, and clear digital information can provide support for patients in relation to planned major surgery. As a complement to previously received oral information and replacement of written information brochures, eHealth applications with both standardised and individually tailored information and instructions illustrated with images or videos can lead to well-informed patients, thereby strengthening patients’ and their relatives’ understanding of and participation in pre- and postoperative self-care. Studies have shown that access to eHealth with customised information can lead to improved self-care [25], and increase personal engagement and participation in as well as responsibility for self-care [26, 27]. This is related to personal health literacy, whereby the individual has the ability to find, understand and use information and services for making health-related decisions and actions for themselves and/or others [28].

In this study, information in eHealth applications was considered important for the healthcare workers for improving patients’ understanding of and active participation in their self-care. As Lindeman et al. [29] also found, our study showed that technology-based interventions must provide accurate and verified information, thereby leading to improved patient safety. A standardised approach to conveying information can, for example, enable patients to reduce symptom burden after surgery. At the same time, patients must be capable of understanding the provided information correctly to perform self-care in a safe manner. In this study, the healthcare workers also worried that too much information in an application may have negative unintended effects. For example, some patients may be unable to read all the information, while others could skip vital information if the amount of the information provided is extensive. Patients’ feedback on the use of eHealth applications in relation to major surgery could further improve pre- and postoperative self-care. Self-care is an area that needs to be highlighted in the development of digital self-care applications in relation to major surgery to achieve safe care and good results.

Accurate information and patients’ knowledge and participation were considered to constitute the foundation for good self-care, and several possible functions in eHealth applications were mentioned as opportunities for improving the safety of pre- and postoperative care. For example, the right information at the right time, also provided in comprehensible language, could help avoid complications and misunderstandings. Push notifications and/or alerts were considered to improve the performance of self-care in a timely manner. Consequently, patients could then manage expected self-care activities more efficiently and with more independence. From a patient safety perspective, these aspects should be considered when developing self-care applications for major surgery. Using a digital application has also been shown to result in a higher degree of patient compliance and fewer cancelled operations, postoperative complications and/or readmissions in neurosurgery [30].

The findings of this study show that self-care applications in relation to major surgery affect the interactions between healthcare workers and patients, which leads to new ways of working in healthcare. Self-care applications can lead to a more efficient use of healthcare resources by saving time and resources for both patients and the healthcare system. For patients, those living far away or with difficulties reaching the hospital would have greater flexibility, which is in line with previous results [17]. Regarding the need to meet patients’ different needs in self-care, our study has revealed health professionals’ doubts over eHealth applications’ capacity to achieve person-centred care, which is perceived to be one of the challenges with the use of a self-care application. The healthcare workers believed that digital solutions may lead to distancing, and there were fears that fewer physical meetings would have a negative impact on the interactions between healthcare workers and patients, as physical meetings create meaning and satisfaction for both parties, also discussed in previous studies [17, 31]. Interestingly, in our study, the healthcare workers believed that digital meetings in real time and patients’ ability to communicate via images could improve two-way communication.

The health professionals believed that patient safety in relation to pre- and postoperative self-care could be increased by targeted eHealth applications. Technology-based interventions with the aim of facilitating access to information can empower patients and family caregivers to take an active role in self-care regardless of time or location [29]. The possibility of communicating with healthcare workers via digital self-registration of postoperative symptoms and via images can also contribute to the early identification of complications and can facilitate continued support for individual patients’ care needs [26, 30]. Gustavell et al. [26] showed that patients who digitally reported symptoms after pancreatic surgery and received support for symptom management by an interactive app experienced greater stability in emotional functions and physical symptoms compared to those who received conventional care. Moreover, the COVID-19 pandemic has also challenged healthcare workers to design safe services for patients.

Shorter hospital stays for inpatient surgery challenge healthcare to streamline perioperative care with maintained or increased quality and, from a person-centred perspective, give support to patients in performing pre- and postoperative self-care. Our study has shown healthcare workers’ awareness that certain patient groups face the risk of digital exclusion due to limited understanding of or access to digital solutions and devices. There may be differences between younger and older people and people with lower socioeconomic status, those who lack the know-how in information seeking and/or those who do not have the ability to use digital tools may suffer further negative consequences [17, 29]. Healthcare workers work using a humanistic approach; therefore, eHealth services should be based on person-centred care [27]. As earlier research has shown, eHealth services cannot replace human relationships and should be considered as a complement to personal meetings [32, 33]. Consequently, healthcare workers need to understand and support pre- and postoperative self-care in a person-centred way and to provide good care on equal terms for the entire population. The challenge ahead is to design digital systems and solutions that ensure information access to healthcare workers and to patients with different conditions and needs so that patient safety can be strengthened. As attitudes may significantly influence eHealth’s acceptance and efficiency of use and function [20], web designers need to work closely with both patients and healthcare workers to include all factors that, based on evidence and experiences, contribute to good healthcare. This knowledge, together with research on patients’ perceptions of eHealth applications, can further contribute insights into designing and implementing digital applications that facilitate patients’ participation in self-care for major surgery. By supporting pre- and postoperative self-care with digital solutions, both patients and the healthcare system can receive various benefits, such as enhanced and safe recovery at home via early recognition of negative signs and symptoms. A self-care application can create new ways of delivering pre- and postoperative person-centred healthcare in an efficient, safe, and cost- and resource-effective way.

### Limitations

The use of semi-structured interviews and analysis in the phenomenographic approach to health and nursing research is aimed at understanding the character of a phenomenon and the different perceptions of that phenomenon [22, 34]. In our study, we used this method to show the variations in healthcare workers’ perceptions of the use of eHealth applications for self-care by patients undergoing major surgery. Most of the participants had no experience of working with eHealth applications in the context of pre- and postoperative selfcare. Despite the digital development during the pandemic, the perceptions found are considered transferable to setting where eHealth is under development. This means that the result is primarily based on the participants’ beliefs and perceptions which, for our study, is a strength. Despite strategically selected participants, there is an imbalance regarding professions and gender, however the distribution of gender and occupational reflect Swedish healthcare settings. Differences may have broadened the results even further. However, the participants were of different ages and genders, had different professional experiences and represented different decision-making levels, all of which strengthened our results. Fewer interviews were conducted than planned, however no new perceptions of the healthcare workers emerged in the last interviews. Another limitation was that the results were based on data collected before the outbreak of COVID-19, which means that healthcare workers’ perceptions may have changed due to the enforced increase of digital care meetings during the pandemic.

## Conclusions

In conclusion, this study improves our understanding of healthcare workers’ perceptions of how eHealth applications can function as tools or obstacles for supporting patients in pre- and postoperative self-care for major surgery. Access to information and instructions, reminder alerts and the possibility of sharing images and thoughts about self-care are some ways of improving patients’ knowledge of self-care, which can lead to enhanced participation and thus improved patient safety. However, certain challenges in future digital development of self-care applications in major surgery must be met to guarantee person-centred care, patient safety and to prevent people being excluded from the care to which they are entitled.

## Data Availability

The datasets used and/or analysed during the current study are available from the corresponding author on reasonable request.
